# Multiple Property Tolerance Analysis for the Evaluation of Missense Mutations

**Published:** 2007-02-24

**Authors:** Tai-Sung Lee, Steven J. Potts, Matthew J. McGinniss, Charles M. Strom

**Affiliations:** 1 Consortium for Bioinformatics and Computational Biology, and Department of Chemistry, University of Minnesota, P.O.Box 14800, Minneapolis, MN 55414; 2 Quest Diagnostics Nichols Institute, 33608 Ortega Highway, San Juan Capistrano, CA 92690

## Abstract

Computational prediction of the impact of a mutation on protein function is still not accurate enough for clinical diagnostics without additional human expert analysis. Sequence alignment-based methods have been extensively used but their results highly depend on the quality of the input alignments and the choice of sequences. Incorporating the structural information with alignments improves prediction accuracy. Here, we present a conservation of amino acid properties method for mutation prediction, Multiple Properties Tolerance Analysis (MuTA), and a new strategy, MuTA/S, to incorporate the solvent accessible surface (SAS) property into MuTA. Instead of combining multiple features by machine learning or mathematical methods, an intuitive strategy is used to divide the residues of a protein into different groups, and in each group the properties used is adjusted.

The results for LacI, lysozyme, and HIV protease show that MuTA performs as well as the widely used SIFT algorithm while MuTA/S outperforms SIFT and MuTA by 2%–25% in terms of prediction accuracy. By incorporating the SAS term alone, the alignment dependency of overall prediction accuracy is significantly reduced. MuTA/S also defines a new way to incorporate any structural features and knowledge and may lead to more accurate predictions.

## Introduction

Computational prediction tools are needed to discover and prioritize candidate human disease alleles from uncharacterized human single nucleotide polymorphisms (SNPs). SNPs are now well known to play a critical but as yet largely uncharacterized role in human disease. However, experimental techniques able to identify deleterious mutations in proteins caused by SNPs are time-consuming and expensive. Although quantitative assessment algorithms do not replace clinically trained experts in diagnostic decisions, they are valuable tools in assisting with a diagnosis (Tchernitchko et al. 2004).

Two categories of algorithms (Saunders and Baker, 2002; Tchernitchko et al. 2004) have been developed recently to predict the mutation effect on protein function: phylogenetic (sequence alignment-based) and structural methods. Phylogenetic methods assume that functionally critical residues are conserved during the evolutionary process and use the phylogenetic information or the degree of conservation for each residue from the alignment of orthologs to predict the mutation effect (Cai et al. 2004; Krishnan and Westhead, 2003; Lau and Chasman, 2004; Mooney and Klein, 2002; Ng and Henikoff, 2001; Tavtigian et al. 2005). The SIFT method and server (Ng and Henikoff, 2001; Ng and Henikoff, 2002; Ng and Henikoff, 2003) is widely used for mutation effect prediction (Tchernitchko et al. 2004).

However, the 20 natural amino acids are intrinsically multi-dimensional in terms of physicochemical properties. For example, lysine (K) and leucine (L) have very similar size (volume) but very different charges and hydrophobicities. Consider the case of a mutation from the wild-type leucine to lysine at a position where phenylalanine (F) and glutamine (Q) have been observed in orthologs. Phenylalanine, leucine, glutamine, and lysine are all similar in size, although very different in other properties. To simultaneously take multiple physicochemical properties into account, Tavtigian et al. used three physicochemical properties to define the “physicochemical” distance of residue types at a given alignment position and predicted the mutation effect based on this definition of distance (Tavtigian et al. 2005). A similar algorithm, MAPP, was developed by Stone and Sidow (Stone and Sidow, 2005) where six physicochemical properties were transformed to orthonormal properties and the physicochemical distance was calculated as a measure to classify mutation effect.

On the other hand, structural approaches attempt to capture the structural or environmental impact of mutation on the target protein residue (Herrgard et al. 2003; Sunyaev et al. 2001; Wang and Moult, 2001; Wang et al. 2003). Attempts to combine both categories of methods are making progress (Bao and Cui, 2005; Ramensky et al. 2002; Saunders and Baker, 2002) by incorporating structural information to complement the alignment-based approaches. Saunders and Baker utilized both classification tree and logistic regression classifier methods to combine multiple predictors, including the SIFT score and other structural features. Ramensky et al, in their PolyPhen server (http://www.bork.embl-heidelberg.de/PolyPhen/), used a set of empirical structure-based rules to predict the mutation effect. Bao and Cui derived several environmental parameters, along with the SIFT score, as the input factors for their support vector machine (SVM) and random forest (RF) methods.

A different approach, PMut by Ferrer-Costa et al. (Ferrer-Costa et al. 2002; Ferrer-Costa et al. 2004), utilizes the neural network learning technique (NN) from a large set of known data to predict the mutation effect in human genes and demonstrates the best prediction accuracy reported so far when the 3D structure information is used. PMut is very powerful for predicting the mutation effect for human genes. However, PMut uses existing mutation data as the base for prediction and, when only considering algorithm, should not be directly compared to other *“ab initio”* methods which only use input sequence alignments and know 3D structures for query sequences.

We here propose a novel strategy for mutation prediction. First we present a sequence alignment-based method, Multiple Properties Tolerance Analysis (MuTA), similar to the method by Tavtigian et al. (Tavtigian et al. 2005) and the MAPP algorithm by Stone and Sidow (Stone and Sidow, 2005) since a set of physicochemical properties are used to measure the degree of conservation for a certain alignment position. The difference is that MuTA calculates the importance for each property independently and selects the most conserved properties as the predictors, while Tavtigian’s method and the MAPP algorithm use all physicochemical properties to calculate the distances.

Secondly, we propose a novel approach, MuTA/S, to incorporate the structural features and the sequence alignment containing evolutionary information. MuTA/S assumes that residue’s functionality and therefore its prediction criteria should depend on its local environment. MuTA/S defines various regions according to their environments and treats each region differently. The concept of region has been seen in methods like PolyPhen (Ramensky et al. 2002; Sunyaev et al. 2003; Sunyaev et al. 2001). In PolyPhen, the concept is used as one of the criteria used for the decision tree, while in MuTA/S it is used to divide a protein into different regions. For each region, the same algorithm but region-optimized parameters are used to perform the prediction.

MuTA already considers local environment effect by selecting different physicochemical properties according to their evolutionary conservation for individual alignment position. However, the evolutionary information from alignment is usually not sufficient, and it is almost impossible to obtain alignments consisting of “perfect” ortholog sequences. As mentioned by Saunders and Baker, structural features, especially SAS, are found useful to increase the prediction accuracy. Unlike Saunders and Baker (Saunders and Baker, 2002) or Bao and Cui’s (Bao and Cui, 2005) methods, in which mathematical treatments or machine learning methods are used to combined various structural features and the SIFT score, MuTA/S groups the residues of the target protein into several regions according to one or a few structural features (in this paper, only solvent accessible area, SAS, is used) and treats each region differently.

## Algorithm

### MuTA Algorithm

The MuTA algorithm first selects the most important physical properties according to the conservation of the properties, and then, for the selected important properties, calculates the deviation of the mutation for a given alignment position. A mutation is determined benign based on whether the deviation is smaller than an empirically determined cutoff or not.

To quantitatively define the degree of deviation for a certain property at an alignment position, we first calculate the mean and the standard deviation of a property from the distribution containing all existing types of amino acids at this position, which we denote as 〈*x_i_*〉 and σ(*x_i_*)*_k_*, respectively:

(1) $$\begin{array}{l} {\langle x_{i}\rangle }_{k}=\frac{\sum_{n}x_{i,n}^{k}}{N}\\ \sigma {\left(x_{i}\right)}_{k}=\sqrt{\frac{\sum_{n}{\left(x_{i,n}^{k}-{\langle x_{i}\rangle }_{k}\right)}^{2}}{N}} \end{array}$$

where *x_i_* denotes the *i*th physicochemical property, *k* is the alignment position, *n* is the index for different types of amino acids occurring at this position, and *N* is the total number of types of amino acids at this position. For example, if there are three type residues at the 100th position of an alignment, A, D, and E, and *x_i_* is the net side-chain charge, 〈*x_i_*〉*_k_* can be calculated as (0 + (−1) + (−1))/3 = − 2/3 and σ(*x_i_*)*_k_* = 0.47140.

MuTA assumes that different physicochemical properties will have different degrees of conservation at different positions. We define the relative importance of the *i*th property *x_i_* at the *k*th alignment position, *I_i_^k^*, is defined as

(2) $$\begin{array}{c} \sigma {\left(x_{i}\right)}_{NAT}=\sqrt{\frac{\sum_{all}{\left(x_{i}-{\langle x_{i}\rangle }_{NAT}\right)}^{2}}{20}}\\ I_{i}^{k}\equiv \frac{\sigma {\left(x_{i}\right)}_{k}}{\sigma {\left(x_{i}\right)}_{NAT}} \end{array}$$

where σ(*x_i_*)*_NAT_* is the standard deviation calculated from the distribution formed by all 20 natural amino acids while σ(*x_i_*)*_k_* is the standard deviation calculated from the distribution formed from the *k*^th^ alignment position. A smaller *I_i_^k^* means the corresponding property is more conserved than the natural distribution of this property and thus thus greater importance.

Given a mutant of certain amino acid type *μ* at the *k*^th^ position, the relative deviation of σ*_μ_^k^* from the above distribution is defined as:

(3) $$\sigma_{\mu }^{k}\equiv \frac{\left|x_{\mu }^{k}-{\langle x_{i}\rangle }_{k}\right|}{\sigma {\left(x_{i}\right)}_{k}+\Delta }$$

where Δ is a small real number. The purpose of Δ is twofold: first to avoid the divided-by-zero error in the case of a totally conserved position where σ(*x_i_*)*_k_* is apparently zero, second to allow some degree of tolerance for a conserved position, e.g. for a conserved position with wild-type residue D, we may change the value Δ so that a mutation to E is considered benign. If Δ = 0.058 is used, a mutation to *K* (charge = +1) in the above example will give σ*_μ_^k^* = |+1−(−2/3)/(0.47140+0.058) = 3.1482.

It is clear that the relative deviation of σ*_μ_^k^* is a measure of deviation of the mutation from the distribution formed by the existing amino acid types at the alignment position. Hence we use it as the criterion to define the mutation as benign or deleterious. A constant threshold, *τ*, is used as:

(4) $$\begin{array}{l} \sigma_{\mu }^{k}\geq \tau \sqrt{M}:\text{deleterious mutation}\\ \sigma_{\mu }^{k}<\tau \sqrt{M}:\text{benign mutation} \end{array}$$

where *M* is the total number of properties. The square-root dependency is determined empirically.

The determination of empirical parameters, Δ and *τ*, is described in the Implementation section.

Thus we have defined a way to measure the deviation of a physicochemical property for a certain mutation from the distribution formed by the alignment.

The MuTA procedure can be summarized as follows: First an alignment containing the query sequence is obtained and a set of physicochemical properties is chosen. For each property at each position, σ(*x_i_*)*_k_*, σ(*x_i_*)*_NAT_*, *I_i_^k^*, and σ*_μ_^k^* are calculated from the above equations. The most important properties are selected according to *I_i_^k^*. In this set of “most important properties,” if there is any property that is considered having deleterious mutation according to Eq.(4), then this mutation is considered deleterious.

In the above equations, we do not consider different weighting of each sequence when alignments are relatively small (no more than 30 sequences). For alignments with large number of sequences ( > 30 sequences), each sequence is given equal weighting so that a single sequence or alignment error will not pollute the results significantly.

### MuTA/S Algorithm

In MuTA/S algorithm, the “region” concept is added into the original MuTA algorithm as follows. For each alignment position, the user can define the region it belongs to. Each region is treated like a separate MuTA system. Different sets of properties can be used and all MuTA parameters are optimized within an individual region. For example, one can define a region in which only charge and side chain size are important so that only these properties are used for MuTA prediction and the cutoff constant will be adjusted.

Regions can be manually defined when sufficient knowledge on the local environment is available. They also can be automatically defined by one or more certain structural properties if they can be calculated from the 3-D structure. In this paper we use relative SAS to classify residues of a protein into four regions:

(5) $$\begin{array}{l} \text{Region }1:\text{Relative SAS}\geq 50\%\\ \text{Region }2:50\%>\text{Relative SAS}\geq 30\%\\ \text{Region }3:30\%>\text{Relative SAS}\geq 20\%\\ \text{Region }4:20\%>\text{Relative SAS}\geq 0\% \end{array}$$

The specific sets of parameters for those regions are described in the System and Method section.

## System and Method

### Property Selection

The selection and exclusion of the appropriate properties are critical to the MuTA approach. It is important for MuTA that the properties are distinct or relatively orthogonal from each other, and that the appropriate number of properties are included. Aaindex v7.0 (Kawashima et al. 1999) lists 516 one dimensional properties and 83 substitution matrices for the 20 natural amino acids. Tomii and Kanehisa (Tomii and Kanehisa, 1996) have analyzed the similarities in 402 amino acid properties using a single-linkage hierarchical cluster analysis, and visualized them using a minimum spanning tree. They demonstrated that amino acid properties can be roughly divided into 6 groups: hydrophobicity, composition, physicochemical properties, beta sheet propensity, alpha helix and turn propensity, and other.

To select properties that were well understood, in frequent use, as distinct from each other as possible, and represent the available properties adequately, we utilized a clustering and visualization technique known as self-organizing maps (SOM). SOM reduces the dimensionality of data through self-organizing neural networks (Kohonen, 1988). Each of the 516 amino acid one dimensional properties was scaled and centered, and then clustered and visualized with the SOM toolbox for Matlab (Vesant, 1999). Each amino acid has its own unique SOM maps for all properties. Properties corresponding to the most dissimilar points in the SOM maps among all amino acids are chosen. The properties chosen are listed in Table 1. Currently, the purpose of the described SOM technique is only to select distinct properties via an easy and visual procedure and should not be treated as a theoretically robust method that may improve the predictive power. We also have not tested the prediction power by different sets of parameters.

Besides the properties used, three empirical parameters also were defined. The first parameter, *τ*, is the threshold to determine when a mutation score is considered damaging. The second parameter, Δ, is a small real number to avoid potential numerical divided-by-zero error. The third parameter, *f*, is the ratio specifying the amount of important properties to be considered. For example, if there are 12 properties and *f* = 4, the first 3 (since 12/4 = 3) most important properties are considered for each position.

In this paper the following parameters are used for MuTA, which are empirically tuned for LacI: *τ* = 2.23, Δ = 0.058, and *f* = 4. For MuTA/S, each region has its own optimized parameter set.

Apparently, physicochemical properties may be highly corrected and their value may be in totally different and unrelated units. Two steps are taken to correct for these two problems. First all properties are normalized: each original property value is subtracted by the mean value and then divided by the standard deviation where the mean and the standard deviation are calculated through the distribution of all 20 amino acids. Principle Component Analysis (PCA) is then used to transfer the normalized properties to mutually orthonormal properties. This set of orthonormal properties was used subsequently in this paper although users can turn the normalization or PCA step off through MuTA’s XML input file. Table 2 shows the contribution from all 10 properties for all principle components.

### Region Definition

SAS was chosen as the region classifier for the MuTA/S algorithm. While there are important exceptions, it has been widely accepted that solvent exposed residues will likely undertake random mutation without affecting the protein function or the binding between the protein and substrates or ligands. Hence we define the first region, Region 1, as consisting of all solvent-exposed residues (relative SAS ≥ 50%). All mutations in this region are considered benign.

Table 3 shows the correlation between relative SAS and the standard deviations for ten properties for LacI. A higher correlation coefficient means the standard deviation for the property is larger when relative SAS is larger. Larger standard deviation for a property means that the property is less conserved or less important, as describe in the previous section aneeerd Eq. (2). Based on this argument, Region 2, consisting of the second most exposed residues (50% > Relative SAS ≥ 30%), uses only a subset of properties. This subset of properties is formed by removing the four properties with highest correlation coefficients in Table 3. The removed properties are Hopp-Woods hydrophobicity, average accessible area in proteins, side chain charge, and solvation energy. A cutoff with larger value, *τ* = 15, is used, which reflects the fact that the more exposed residues should have higher cutoff to be considered deleterious. Region 3, which consists of residues with relative SAS sitting between 20% and 30%, uses a lower cutoff, *τ* = 9.5. Region 4, consisting of buried residues, uses the lowest cutoff, *τ* = 2.13, and all properties are selected (*f* = 1). The parameters for each region are manually tuned against LacI and Lysozyme with manually curated alignments.

The original MuTA parameters, empirically tuned for LacI, are *τ* = 2.23, Δ = 0.058, and *f* = 4. The definition of each region and their parameters are listed as follows:

(6) $$\begin{array}{l} \text{Region }1:\text{Relative SAS}\geq 50\%:\\ \text{All mutations are treated as benign}.\\ \text{Region }2:50\%>\text{Relative SAS}\geq 30\%:\\ \tau =15,\Delta =0.058,\text{and }f=2;\\ \text{a subset of properties}\text{.}\\ \text{Region }3:30\%>\text{Relative SAS}\geq 20\%:\\ \tau =9.5,\Delta =0.058,\text{and }f=1;\\ \text{all properties}\text{.}\\ \text{Region }4:20\%>\text{Relative SAS}\geq 0\%:\\ \tau =2.13,\Delta =0.058,\text{and }f=1;\\ \text{all properties}\text{.} \end{array}$$

### Protein Systems

Three protein systems were used in this study, LacI (Markiewicz et al. 1994), T4-Lysozyme (Rennell et al. 1991), and HIV-1 protease (Loeb et al. 1989). The structures used to calculate SAS are the following Protein Data Bank (Berman et al. 2000) (PDB) structures: 1EFA for LacI, 3LZM for T4 lysozyme, and, 1DIF for HIV-1 protease. The SAS for HIV-1 protease and LacI were calculated from dimers, while SAS for Lysozyme was calculated from single protein chain.

SAS values were calculated by the GetArea server (http://www.scsb.utmb.edu/cgibin/get a form.tcl) at Sealy Center for Structural Biology, University of Texas Medical Branch (Fraczkiewicz and Braun, 1998). The default atomic van der Waals parameters and a standard 1.4 Ǻ solvent probe radius were used.

All SIFT results reported here were calculated by the SIFT program downloaded from the SIFT server (http:http://blocks.fhcrc.org/sift/SIFT.html). All experimental results are also taken from the SIFT server. The MAPP program was downloaded from http://mendel.stanford.edu/SidowLab/downloads/MAPP/MAPP.html.

The MuTA (including MuTA/S) program was implemented in ANSI C++ with Standard Template Library (STL) and Template Numerical Toolkit (TNT) for Linear Algebra (Barrett et al. 1994). The input parameters and output results are in XML format and are extensible and portable. The program runs on Window XP SP2 and Linux RedHat 9.0. The compilers used are Microsoft Visual C++ 6.0 SP6 (Win32) and Intel C++ 9.0 (Linux). The test cases were performed on a Dell D600 notebook computer with a 1.7GHz Pentium-M CPU and 2 GB RAM. A web portal running MuTA and MuTA/S program is available to Quest Diagnostics’ internal usage and will soon be available for public access.

## Results and Discussion

LacI and T4-Lysozyme were chosen for tuning and benchmarking for both MuTA and MuTA/S. The experimental data are taken from the SIFT server (http://blocks.fhcrc.org/sift/SIFT.html). Since NR and SwissProt databases keep been updated and alignments from those databases will be different at different time, our SIFT results are slightly different from the original reported SIFT results. The term “benign” used in this paper has the same meaning as the term “tolerant” in the SIFT paper by Ng and Henikoff (Ng and Henikoff, 2001). However, the term “deleterious” here refers to the mutation with strong deleterious effect while the same term used in the SIFT paper means all non-tolerant mutations. The definition used here has been used recently (Stone and Sidow, 2005). These differences lead to different number of data points and different results for SIFT in our result.

MuTA and MuTA/S was manually tuned for these alignments hence the results can be considered as “training data set”. Table 4 shows the results from SIFT, MAPP, MuTA, and MuTA/S with different regions from human expert curated alignments. Similar performance is seen for MuTA, SIFT and MAPP. Nevertheless, MuTA/S gives better results when more regions are used. When all four regions are used, the overall prediction percentage of MuTA/S is significant better than SIFT and MuTA. SIFT, in multiple studies, has given the good results to date for sequence-based prediction (Saunders and Baker, 2002; Tchernitchko et al. 2004). To our knowledge, the prediction percentages for LacI and Lysozyme by MuTA/S are the best results reported in the literature so far if empirical learning methods, such as PMut (Ferrer-Costa et al. 2002; Ferrer-Costa et al. 2004), are not considered. Further examining the results of Lysozyme, we found that most of false positive results (experiment = benign; prediction = deleterious) are from highly buried and totally conserved residues. It is almost impossible for an alignment-based prediction method to correctly predict such mutations, unless detailed atomic-level information and/or interaction of this type of residues can be used in the prediction.

In addition to the above training data set, we performed MuTA/S analysis on LacI, Lysozyme, and HIV-1 protease (Loeb et al. 1989) with different alignments. The alignments, taken from the SwissProt database (Bairoch and Apweiler, 2000) and NCBI’s non-redundant database (NR, http://www.ncbi.nlm.nih.gov/) through the SIFT server (http://blocks.fhcrc.org/sift/SIFT.html), should be considered as “test data sets” since MuTA/S is not optimized against them. Those three sets of data (LacI, Lysozyme, and HIV-1 Protease) have been widely used as the benchmark sets of mutation effect prediction. The results are listed in Table 5, Table 6, and Table 7, respectively. In addition to these three sets of data, results for two highly interested genes, Cystic fibrosis transmembrane conductance regulator, CFTR (Riordan et al. 1989), and Glucose-6-phosphate dehydrogenase, G6PD (Kwok et al. 2002), are listed in Table 8. For all data sets, MuTA/S consistently outperforms SIFT and MuTA by 2% to 25%. We were not able to perform high throughput runs via PolyPhen or PMut web interfaces hence no direct comparison to MuTA/S can be made.

The main features of MuTA are that multidimensional predictors, which are physicochemical properties, are used rather than a single predictor, and that the selection of the predictors is based on the conservation of the predictors at the specific position. The use of multiple physicochemical properties as predictors combined with the position-specific selection of the predictors has at least three advantages: Firstly, the prediction is more reliable since different predictors are used for different environments. Secondly, for a specific position, the predictor(s) chosen reflect the importance of certain physicochemical properties at this position. This information can be further examined and rationalized with structural or other types of data. Thirdly, the choice of properties can be structural properties, e.g. at a given alignment position, solvent accessible areas for all sequences if their structures are known or can be modeled. Thus structure-specific properties can be used, while only amino acid type-specific data can be used in SIFT or other similar approaches.

All major sequence alignment-based prediction methods, including SIFT, PMut, PolyPhen, or MAPP, ignore the inter-residue interaction, at least explicitly. The prediction of a mutation effect on covariant residues (Clarke, 1995) is very difficult, if not impossible. In such cases, successful prediction may require molecular dynamic or free energy perturbation simulations at the atomic level to understand the detailed interactions between residues, or co-variance analysis using the sequence alignment to assess the dependency rules for residues. Local conformational changes for sequences to sequences in the same alignment will void conservation analysis-based mutation prediction. Again, other algorithms or simulation tools are needed in such cases.

Because sequence-based methods, such as SIFT and MuTA, highly depend on accurate sequence alignments, an alignment consisting of widely spread ortholog sequences for the same function will be ideal. In practice, it is difficult to have such sets of sequences. Automated genome-wide methods would benefit substantially if a way to distinguish and extract information from ortholog and paralog sequences is defined and employed in the conservation analysis. Because the goal is for these algorithms to support human experts making diagnostic testing decisions, we believe that careful manual preparation of alignments is a vital component for providing a useful sequencing assay service. This is a different approach than offered by web-based tools that operate across the entire human genome, in which alignments are generated automatically.

Saunders and Baker concluded that accurate mutation prediction requires sufficient evolutionary information, but structural information may increase the accuracy when there is lack of evolutionary information (Saunders and Baker, 2002). Our results in Table 5, Table 6, and Table 7 clearly confirm their conclusions, although the number of sequences seems not a determining factor. The average Shannon’s Entropy (Shannon, 1984), *I*, for each alignment, which is a measure of the sequence divergence, is also listed in Table 5, Table 6, and Table 7. Our results show that alignments with enough sequence divergence are critical. SIFT and MuTA results using SwissProt alignments are consistently better than the results using NR alignments, which is expected since a sequence search against the SwissProt database would return more diverged sequences than the NR database due to the fact that NR has much more highly-similar sequences. When only NR sequences are used, SIFT and MuTA usually predict relatively poorly.

MuTA/S, on the other hand, not only outperforms SIFT or MuTA but is more stable against different alignments. For example, Table 5 shows that the maximum difference in prediction accuracy is around 23% for LacI when SIFT is used (50.26% vs. 72.93%), while it goes down to around 7% using MuTA/S (70.68% vs. 77.86%). For the three protein systems the worst prediction accuracy from MuTA/S is 69.81% while the lowest accuracy from SIFT and MuTA is as low as 50%. An accuracy of 50% means an incorrect prediction half of the time, or almost no distinguishing prediction power.

In commercial diagnostic testing, usually a limited number of gene tests are performed in very large volumes. The alignments must be correct, so hand curated alignments are both practical and critical to obtaining accurate prediction results. The alignment of sequences can easily be automated with many different alignment algorithms generally producing very similar results. However, the choice of which orthologs and paralogs can currently be done with better results by a human expert than a computer. For example, depending on the degree a particular protein function is conserved, it may be appropriate for one protein system to include conserved orthologs across all vertebrates, but in another system to include only mammals. The inclusion of paralogs (or paralogous domains) also frequently needs to be evaluated for each system. We selected sequences and performed curated alignments on only a small set of genes for clinical diagnostic purposes. Those curated alignments are for highest possible alignment-based preditions.

However, for an automated prediction server for any gene, the greater stability of results against different alignments is critical. With genome-wide automated prediction, a user normally would supply an input sequence and probably request automatic sequence search against public sequence databases, such as SwissProt or NR databases, to build the alignment. In such cases, the quality of the input alignments could be not ideal for sequence alignment-based mutation predictions. A stable method like MuTA/S can at least give users reasonable results, although, unlike SIFT, currently MuTA and MuTA/S contain only the prediction algorithm and do not provide automatically sequence search and alignment functions, which can be easily done be various available tools such Psi-BLAST. Another alternative approach has seen in PMut (Ferrer-Costa et al. 2002; Ferrer-Costa et al. 2004), where a large set of data of a certain gene is preprocessed and the prediction is based on the neural network learning results hence less alignment-dependent results would be expected.

For MuTA/S, we already mentioned that it is not always true that solvent exposed residues will undertake random mutation without affecting the protein function or the binding between the protein and substrates or ligands. The Region 1 definition used in this paper considers all exposed residues (relative SAS ≥ 50%) benign, which is clearly not correct in some cases, although we found that this rule correctly predicts benign mutations for more than 80% of cases. To further increase the prediction accuracy, this issue must be addressed. The exceptions of the Region 1 rule could be: 1. the residues could play the role like a “gatekeeper” for controlling or specifying the substrates/ligands entrance or exit; 2. they could be important for protein-protein interactions; 3. the residues could be in fact not solvent-exposed *in vivo*; 4. other unknown reasons. To address those exceptions, detailed biochemical knowledge may be necessary. Furthermore, extra caution should be taken when calculating SAS for a protein. For example, SAS from HIV-1 protease dimmer with substrate/ligand should be used, not SAS from an HIV-1 protease monomer. Also, our current implementation is not able to deal with multiple structures for one alignment. One possible solution is that every alignment position is assigned to its SAS region according to the maximum SAS in the structures, since high SAS probably means the alignment position is more tolerate to the mutation.

The concept of region in MuTA/S can be extended beyond SAS classification. For example, consider a ligand binding site region where the properties of size and charge are important. Only these two properties could be used for mutation effect prediction within this region. Hence we define a way to incorporate structural and/or mechanism knowledge into prediction methods. This concept could be applied to protein systems where substantial structure-function knowledge is available and will lead to highly accurate prediction for specific protein systems. Such approach will improve prediction results further in well-studied systems. It also can be applied to other sequence-based approaches, such as SIFT or MAPP: the empirical parameters can be optimized for different regions and improved results should be expected.

In summary, we present the MuTA algorithm and its extension, the MuTA/S algorithm. MuTA provides a framework for mutation prediction methods while MuTA/S is based on this framework and utilizes SAS information into the prediction. Tests on LacI, Lysozyme, and HIV-1 protease show that MuTA/S significantly improves the prediction accuracy and reduces the alignment dependency. The approach of MuTA/S also provides the possibility to incorporate other structural or mechanism knowledge to the mutation effect prediction.

## Figures and Tables

**Table 1:** The physicochemical properties used for the 20 natural amino acid residues (single letter codes are shown). The units are not shown since they are irrelevant in the calculations.

	A	C	D	E	F	G	H	I	K	L
Kyte-Doolittle Hydrophobicity	1.8	2.5	−3.5	−3.5	2.8	−0.4	−3.2	4.5	−3.9	3.8
Hopp–Woods Hydrophobicity	−0.5	−1	3	3	−2.5	0	−0.5	−1.8	3	−1.8
pKa value for free amino acid carboxylate	2.3	1.8	2	2.2	1.8	2.4	1.8	2.4	2.2	2.4
Number of sulfur atoms in amino acid	0	1	0	0	0	0	0	0	0	0
Average accessible area in proteins	31.5	13.9	60.9	72.3	28.7	25.2	46.7	23	110.3	29
Volume	88.6	108.5	111.1	138.4	189.9	60.1	153.2	166.7	168.6	166.7
Side Chain Charge	0	0	−1	−1	0	0	0	0	1	0
Polarity	0	1.48	49.7	49.9	0.35	0	51.6	0.13	49.5	0.13
Aromatic residue	0	0	0	0	1	0	0	0	0	0
Solvation Energy	1.93	−1.24	−6.7	−6.47	−0.89	1.00	−10.25	2.15	−4.29	2.28

	***M***	***N***	***P***	***Q***	***R***	***S***	***T***	***V***	***W***	***Y***

Kyte-Doolittle Hydrophobicity	1.9	−3.5	−1.6	−3.5	−4.5	−0.8	−0.7	4.2	−0.9	−1.3
Hopp-Woods Hydrophobicity	−1.3	0.2	0	0.2	3	0.3	−0.4	−1.5	−3.4	−2.3
pKa value for free amino acid carboxylate	2.3	2	2	2.2	1.8	2.1	2.6	2.3	2.4	2.2
Number of sulfur atoms in amino acid	1	0	0	0	0	0	0	0	0	0
Average accessible area in proteins	30.5	62.2	53.7	74	93.8	44.2	46	23.5	41.7	59.1
Volume	162.9	114.1	112.7	143.8	173.4	89	116.1	140	227.8	193.6
Side Chain Charge	0	0	0	0	1	0	0	0	0	0
Polarity	1.43	3.38	1.58	3.53	52	1.67	1.66	0.13	2.1	1.61
Aromatic residue	0	0	0	0	0	0	0	0	1	1
Solvation Energy	−1.49	−9.71	2.00	−9.41	−10.91	−5.11	−5.01	1.98	−5.91	−6.13

**Table 2:** The principle components (PC*n*) used in the calculations. The entries (in percentage) are the contributions from different properties.

	PC1	PC2	PC3	PC4	PC5	PC6	PC7	PC8	PC9	PC10
Kyte-Doolittle Hydrophobicity	20.85	0.18	0.68	1.32	0.02	15.36	3.50	1.23	43.16	13.68
Hopp-Woods Hydrophobicity	17.57	9.89	0.09	0.44	0.82	4.34	11.42	0.16	27.43	27.85
pKa value for free amino acid carboxylate	4.45	0.47	41.16	3.93	37.74	4.31	0.02	7.60	0.31	0.00
Number of sulfur atoms in amino acid	2.30	2.03	51.54	0.23	30.00	5.80	7.04	0.57	0.08	0.41
Average accessible area in proteins	19.87	1.86	2.07	4.91	1.56	0.16	13.63	20.32	0.01	35.60
Volume	0.00	39.56	0.39	0.39	16.61	11.19	3.96	13.47	0.97	13.45
Side Chain Charge	18.00	0.03	0.83	0.66	4.51	30.26	3.37	32.37	2.22	7.73
Polarity	15.04	4.33	0.82	4.78	0.83	27.04	26.75	0.33	19.64	0.44
Aromatic residue	1.44	36.68	0.09	8.09	1.97	0.21	29.86	15.58	5.98	0.10
Solvation Energy	0.48	4.95	2.31	75.25	5.94	1.33	0.45	8.36	0.19	0.74

**Table 3:** The correlation coefficients between the relative SAS and the standard deviation of ten physicochemical properties.

Kyte-Doolittle Hydrophobicity	0.29
Hopp-Woods Hydrophobicity	0.45
pKa value for free amino acid carboxylate	0.39
Number of sulfur atoms in amino acid	−0.21
Average accessible area in proteins	0.54
Volume	0.28
Side Chain Charge	0.54
Polarity	0.42
Aromatic residue	−0.12
Solvation Energy	0.52

1 The standard deviations of properties are calculated from the human curated alignment of LacI.

**Table 4:** Comparison of prediction accuracy for SIFT, MAPP, MuTA, and MuTA/S with different regions.

	Lysozyme			LacI		

	Benign	Deleterious	Prediction Accuracy	Benign	Deleterious	Prediction Accuracy
SIFT	864/1377	169/175	79.66	1764/2267	767/1166	71.80
MAPP	826/1377	170/175	78.57	1795/2267	834/1166	75.35
MuTA	790/1377	169/175	76.97	1819/2267	762/1166	72.79
R1	957/1377	166/175	82.18	1901/2267	753/1166	74.22
R1+R2	1079/1377	166/175	86.61	1964/2267	725/1166	74.41
R1+R2+R3	1112/1377	166/175	87.81	2000/2267	707/1166	74.43
R1+R2+R3+R4	1075/1377	174/175	88.75	1925/2267	788/1166	76.25

1 For the “Benign” entries, the first number is the number of benign predictions and the second number is the total number of experimentally confirmed benign mutations. “Deleterious” entries have a similar meaning but are for deleterious mutations.

2 The entries of “Prediction Accuracy” are the prediction percentages calculated by averaging the benign and deleterious percentages.

3 R*n* means MuTA/S with Region *n*, defined in the System and Method section.

**Table 5:** Prediction results from SIFT, MuTA, and MuTA/S using different alignments for LacI. Percentage entries are in the format of “overall percentage (benign percentage, deleterious percentage)”. The overall percentage is the average of the benign percentage and the deleterious percentage.

LacI	n	I	SIFT	MuTA	MuTA/S
Manual	9	1.01	71.80 (77.81,65.78)	72.79 (80.24,65.35)	76.25 (84.91,67.58)
SwissProt	6	0.84	71.58 (79.00,64.15)	72.22 (68.02,76.42)	77.52 (79.31,75.73)
NR.7	7	0.01	49.47 (0.75,98.20)	49.65 (1.28,98.03)	70.68 (51.83,89.54)
NR.14	14	0.01	50.26 (0.53,100)	49.61 (1.01,98.20)	70.70 (51.52,89.88)
NR.29	29	0.54	72.93 (64.12,81.73)	72.83 (69.08,76.59)	76.94 (77.81,76.07)
Swiss+NR.29	34	0.71	72.2 (68.58,75.81)	71.96 (68.37,75.56)	77.86 (77.33,78.39)

1 Manual is human-curated alignment.

2 SwisProt is the alignment from PsiBLAST search results from the SwissProt database.

3 NR is the alignment from PsiBLAST search results from NCBI’s non-redundant database. NR.7 stands for the first 7 sequences.

4 Swiss+NR is the alignment combined with the SwissProt and NR sequences. Redundant sequences are not removed.

5 n is the number of sequences in the alignment, including the query sequence.

6 I is the average entropy.

**Table 6:** Prediction results from SIFT, MuTA, and MuTA/S using different alignments for Lysozyme. Percentage entries are in the format of “overall percentage (benign percentage, deleterious percentage)”. The overall percentage is the average of the benign percentage and the deleterious percentage.

Lysozyme	n	I	SIFT	MuTA	MuTA/S
Manual	8	0.71	79.66 (62.75,96.57)	76.97 (57.37,96.57)	88.75 (78.07,99.43)
SwissProt	3	0.69	75.61 (68.36,82.86)	69.87 (44.88,94.86)	85.19 (75.53,94.86)
NR.80	80	0.04	50.44 (0.87,100)	51.65 (9.01,94.29)	77.04 (57.52,96.57)
NR.165	165	0.05	50.29 (0.58,100)	54.82 (13.07,96.57)	78.31 (59.48,97.14)
NR.329	329	0.13	56.21 (12.42,100)	61.92 (29.56,94.29)	81.70 (67.97,95.43)
Swiss+NR.329	331	0.15	58.50 (16.99,100)	63.87 (36.31,91.43)	82.48 (71.24,93.71)

1 Manual is human-curated alignment.

2 SwisProt is the alignment from PsiBLAST search results from the SwissProt database.

3 NR is the alignment from PsiBLAST search results from NCBI’s non-redundant database. NR.80 stands for the first 80 sequences.

4 Swiss+NR is the alignment combined with the SwissProt and NR sequences. Redundant sequences are not removed.

5 n is the number of sequences in the alignment, including the query sequence.

6 I is the average entropy.

**Table 7:** Prediction results from SIFT, MuTA, and MuTA/S using different alignments for HIV-1 protease. Percentage entries are in the format of “overall percentage (benign percentage, deleterious percentage)”. The overall percentage is the average of the benign percentage and the deleterious percentage.

HIV-PR	n	I	SIFT	MuTA	MuTA/S
Manual	48	1.55	62.71 (84.55,40.88)	68.24 (100,36.48)	69.81 (100,39.62)
HV1	20	0.25	57.03 (15.32,98.74)	59.06 (22.52,95.60)	76.12 (79.28,72.96)
HV1HV2	30	0.54	69.88 (50.45,89.31)	67.28 (54.05,80.50)	76.89 (86.49,67.30)
SwissProt	43	0.78	79.61 (71.17,88.05)	75.45 (81.08,69.81)	77.62 (95.5,59.75)
NR.50	50	0.04	54.05 (8.11,100)	56.71 (15.32,98.11)	77.69 (79.28,76.10)
NR.400	400	0.05	51.80 (3.60,100)	59.92 (26.13,93.71)	76.79 (77.48,76.10)
NR.100	100	0.04	51.80 (3.60,100)	57.62 (17.12,98.11)	78.32 (79.28,77.36)
Swiss+NR.400	442	0.19	70.32 (45.05,95.6)	73.58 (60.36,86.79)	80.27 (90.09,70.44)

1 Manual is human-curated alignment.

2 HV1 is the alignment consisting of only HIV-type 1 sequences from the NR database.

3 HV1HV2 is the alignment consisting of HIV-type 1 and type 2 sequences from the NR database.

4 SwisProt is the alignment from PsiBLAST search results from the SwissProt database.

5 NR is the alignment from PsiBLAST search results from NCBI’s non-redundant database. NR.50 stands for the first 50 sequences.

6 Swiss+NR is the alignment combined with the SwissProt and NR sequences. Redundant sequences are not removed.

7 n is the number of sequences in the alignment, including the query sequence.

8 I is the average entropy.

**Table 8:** Prediction results from SIFT, MuTA, and MuTA/S for CFTR and G6PD. Entries are in the format of “overall percentage (benign percentage, deleterious percentage)”. The overall percentage is the average of the benign percentage and the deleterious percentage.

	n	SIFT	MuTA	MuTA/S
CFTR	61	65.47 (35.29,95.65)	62.02 (58.82,65.22)	78.01 (64.71,91.3)
G6PD	34	53.67 (20.00,87.34)	60.81 (52.00,69.62)	67.45 (64.00,70.89)

1 Alignments are human-curated.

2 The structure for CFTR is taken from PDB ID:2F9Q; the structure for G6PD is taken from PDB ID:1QKI.
